# P-1182. Impact of Standard Infection Prevention Measures, Including Contact Precautions, on *Serratia marcescens* Outbreaks in the Neonatal ICU

**DOI:** 10.1093/ofid/ofae631.1368

**Published:** 2025-01-29

**Authors:** Kelsey L Rowe, Geoffrey Ikpeama, Ashley Lloyd, Louise Jadwisiak, Melissa Riley, Barbara Warner, Carly Wheeler, Paige Arndt, Keely Watts, Latoya Daughrity, Stephanie A Fritz, Patrick J Reich

**Affiliations:** Washington University School of Medicine, Saint Louis, Missouri; St. Louis Children's Hospital, St. Louis, Missouri; St. Louis Children's Hospital, St. Louis, Missouri; St. Louis Children's Hospital, St. Louis, Missouri; Washington University School of Medicine, Saint Louis, Missouri; Washington University School of Medicine, Saint Louis, Missouri; St. Louis Children's Hospital, St. Louis, Missouri; St. Louis Children's Hospital, St. Louis, Missouri; St. Louis Children's Hospital, St. Louis, Missouri; St. Louis Children's Hospital, St. Louis, Missouri; Washington University School of Medicine, Saint Louis, Missouri; Washington University School of Medicine, Saint Louis, Missouri

## Abstract

**Background:**

*Serratia marcescens* outbreaks attributed to lapses in hand hygiene, contaminated sinks or drains, and other sources of environmental contamination have been observed in the neonatal intensive care unit (NICU).^1^*S. marcescens* outbreaks were identified in the St. Louis Children’s Hospital (SLCH) NICU, a level IV NICU with 150 beds, in 2019 and 2023.

NICU Location and Infant Body Sites of Serratia marcescens Infections

**Figure:**
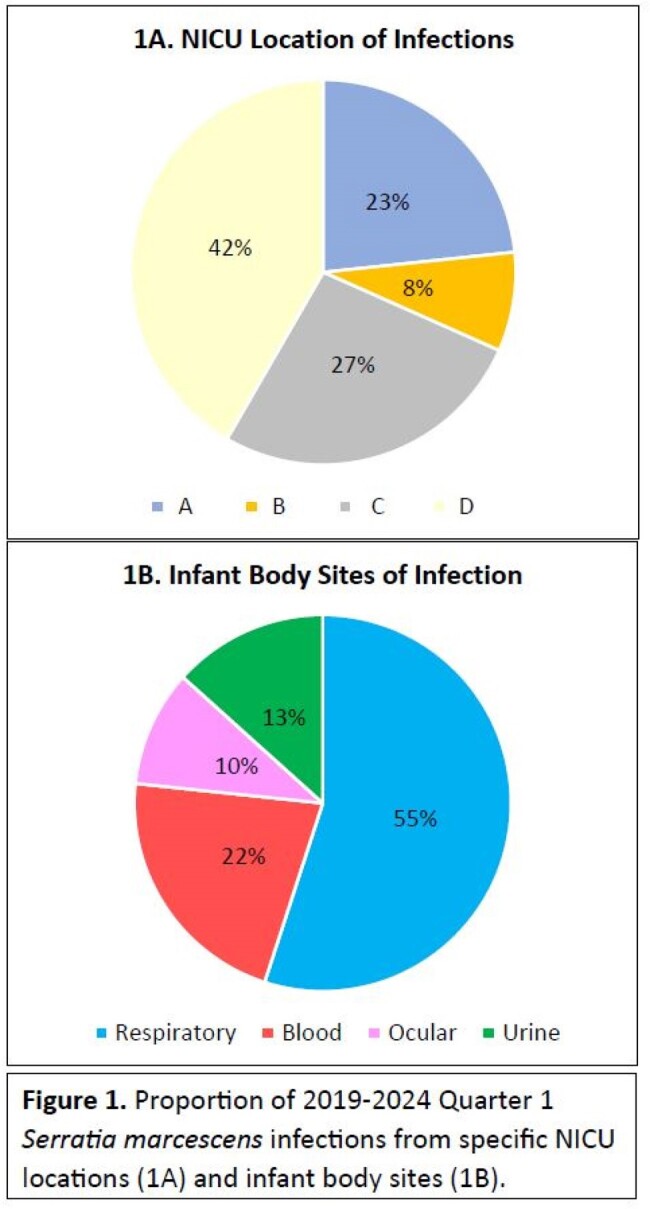

Proportion of 2019-2024 Quarter 1 Serratia marcescens infections from specific NICU locations (1A) and infant body sites (1B).

**Methods:**

A detailed review of NICU *S. marcescens* infections was performed and quarterly infection rates per 1,000 patient-days were followed.

The following response measures were implemented in ­­2020:

1. Contact isolation precautions (CP) for infants with *S. marcescens* infections.

2. Education provided to NICU staff:

a. Impact of *S. marcescens* infections in infants.

b. Potential sources of *S. marcescens* in the NICU.

c. Importance of standard infection prevention measures, including hand hygiene, nail hygiene, isolation precautions, and environmental cleaning.

3. Cleaning or exchange of NICU sink aerators.

4. Installation of splash guards next to sinks in medication rooms.

The following response measures were implemented in 2023:

5. Steps 1-3 above were re-implemented.

6. Sterile water was substituted for tap water during infant baths.

7. Whole genome sequencing was performed on seven *S. marcescens* isolates. A cutoff of >30 allelic differences between isolates indicated genetically unrelated strains.

Annotated NICU Serratia marcescens Infection Rate

**Figure:**
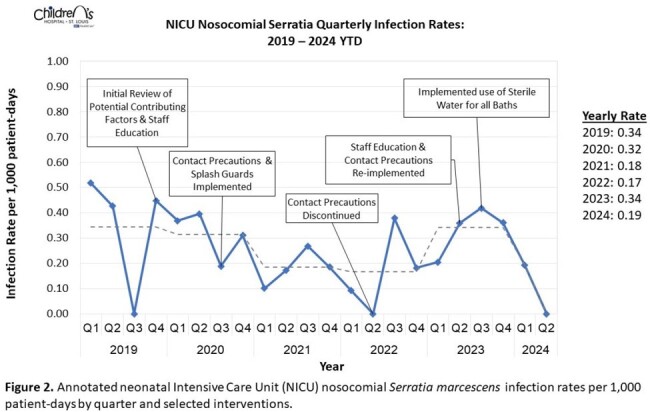

Annotated neonatal intensive care unit (NICU) nosocomial Serratia marcescens infection rates per 1,000 patient-days by quarter and selected interventions. The dotted grey line indicates the average annual infection rate.

**Results:**

Geographic clustering was identified in specific NICU locations (Figure 1A). The most common body site of infection was the respiratory tract, followed by bloodstream (Figure 1B). A sustained decrease in *S. marcescens* infection rates was observed following 2020 response measures (Figure 2). As such, CP for infants with *S. marcescens* was paused. In 2023, *S. marcescens* infection rates again doubled and re-implementation of interventions resulted in decreased infection rates. Isolates were genetically unrelated based on whole genome sequencing (Figure 3).

Whole Genome Sequencing Results

**Figure:**
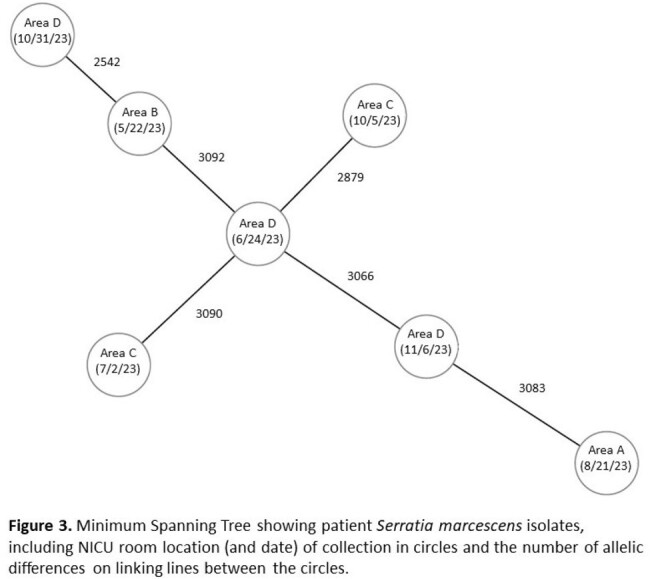

Minimum Spanning Tree showing patient Serratia marcescens isolates, including NICU room location (and date) of collection in circles and the number of allelic differences on linking lines between the circles.

**Conclusion:**

Standard infection prevention measures, including CP, and mitigation of potential water exposure sources resulted in decreased *S. marcescens* infection rates following two separate outbreaks in the NICU. Despite temporal and geographic clustering, isolates were genetically unrelated.

**Disclosures:**

**All Authors**: No reported disclosures

